# DEApp: an interactive web interface for differential expression analysis of next generation sequence data

**DOI:** 10.1186/s13029-017-0063-4

**Published:** 2017-02-03

**Authors:** Yan Li, Jorge Andrade

**Affiliations:** Center for Research Informatics, University of Chicago, Knapp Center for Biomedical Discovery, 900 East 57th St., Chicago, 60637 USA

**Keywords:** Next Generation Sequence (NGS), Differential Expression (DE) analysis, Web interface, R, Shiny, Genomics

## Abstract

**Background:**

A growing trend in the biomedical community is the use of Next Generation Sequencing (NGS) technologies in genomics research. The complexity of downstream differential expression (DE) analysis is however still challenging, as it requires sufficient computer programing and command-line knowledge. Furthermore, researchers often need to evaluate and visualize interactively the effect of using differential statistical and error models, assess the impact of selecting different parameters and cutoffs, and finally explore the overlapping consensus of cross-validated results obtained with different methods. This represents a bottleneck that slows down or impedes the adoption of NGS technologies in many labs.

**Results:**

We developed DEApp, an interactive and dynamic web application for differential expression analysis of count based NGS data. This application enables models selection, parameter tuning, cross validation and visualization of results in a user-friendly interface.

**Conclusions:**

DEApp enables labs with no access to full time bioinformaticians to exploit the advantages of NGS applications in biomedical research. This application is freely available at https://yanli.shinyapps.io/DEAppand https://gallery.shinyapps.io/DEApp.

## Background

Next Generation Sequencing (NGS) technologies provide significant advantages over its predecessors for the study of complex genomic features associated with human disease in the filed of biomedical research [1–5]. Significant progress have been made for the analysis of NGS data, this includes improvement on the accuracy of reads alignment for highly repetitive genomes, precise quantification of transcripts and exons, analysis of transcript isoforms and allele specific expressions. However, large-scale data management and the complexity of downstream differential expression (DE) analysis still remain a challenge that restrains the use of NGS technologies.

Even though several open source analysis tools are currently available for the DE analysis of count based sequence data, each tool implements a different algorithm, uses a specific statistical model, and is susceptible to a specific error model. Changing the models or the parameters used in a particular tool often results in dramatic changes on the detected DE features. Additionally, the use and manipulation of available bioinformatics tools requires of computer programing and command line knowledge that is not always present in many biomedical labs.

To address these challenges, we have developed DEApp, a web based application designed to aid with data manipulation and visualization when performing DE analysis on count-based summaries from sequencing data. DEApp can be used to perform differential gene expression analysis using read counts from RNA-Seq data, differential methylated regions analysis using read counts from ChIP-Seq data, and differential expression small RNA analysis using counts from small RNA-Seq data. DEApp is a self-oriented web based user friendly graphical interface, which enables users lacking of sufficient computational programing knowledge to conduct and cross-validate DE analysis with three different methods: edgeR [6], limma-voom [7], or DESeq2 [8].

## Implementation

DEApp is developed in R [9] with Shiny [10]. It has been configured and launched at the RStudio Shinyapps.io cloud server, and can be easily accessed using any operating system, without requiring any software installation. With DEApp users are able to upload their data, evaluate the effect of model selections, interactively visualize parameter cutoffs modifications, and finally cross validate the analysis results obtained from different methods. DEApp implements the entire computational analysis on the background server, and display results dynamically on the graphical web interface. All result files and figures displayed on the interface can be saved locally.

## Results and discussion

**Fig. 1:**
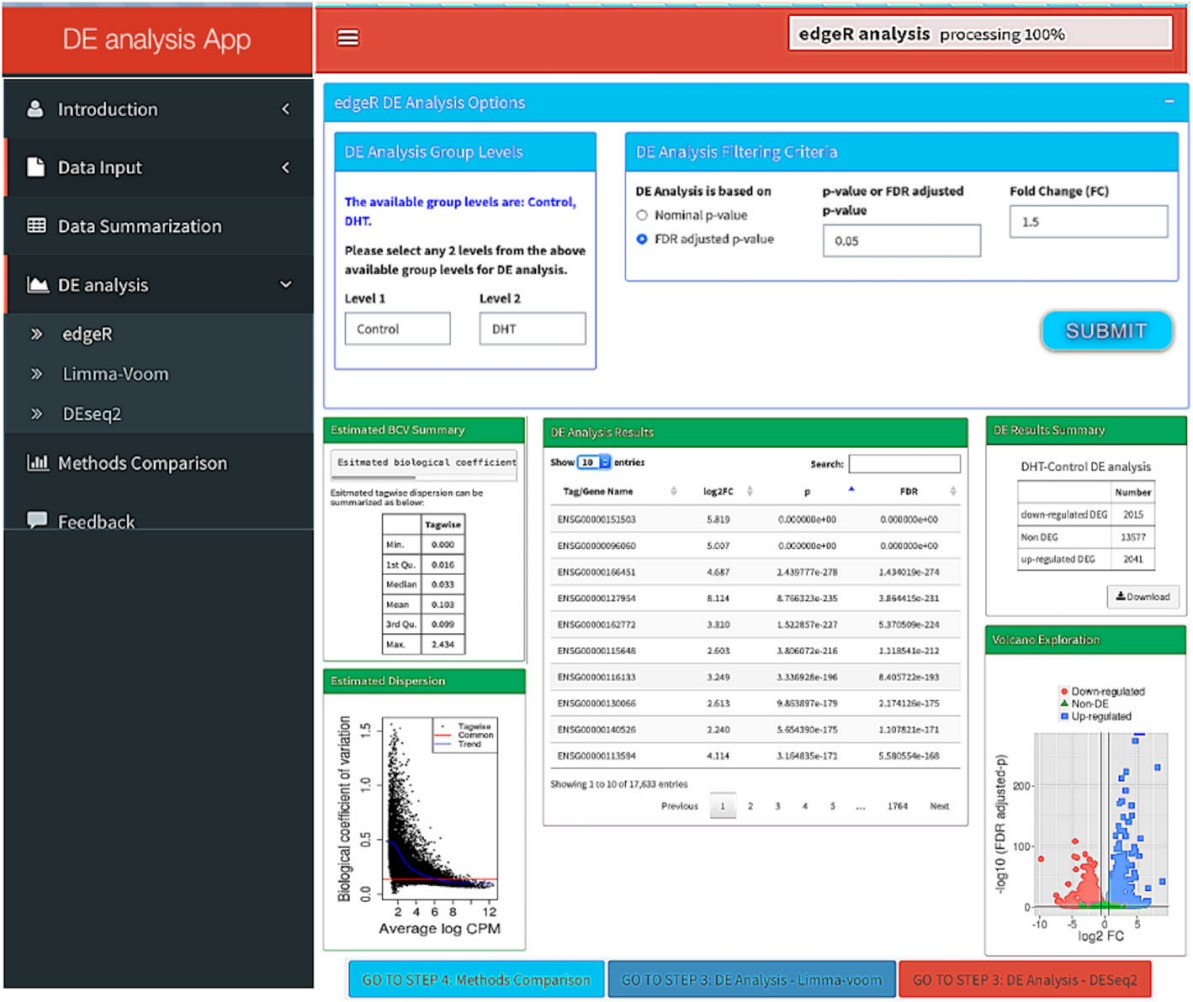
Illustration of DEApp web interface, edgeR analysis section. The *left black dashboard sidebar* illustrates the analysis workflow; the *top blue box panel* of each analysis section shows the input panels for various DE cutoffs; the *green box panels* show the analysis results and visualizations

DE analysis with DEApp is performed in 4 steps: ‘Data Input’, ‘Data Summarization’, ‘DE analysis’, and ‘Methods Comparison’. Figure 1 shows an example of the graphical web interface of DEApp with edgeR for DE analysis. Two files are required as input data for this application, the ‘Raw Count Data’ and ‘Meta-data Table’. The ‘Raw Count Data’ contains summarized count results of all samples in the experiment, and the ‘Meta-data Table’ contains summarized experimental design information for each sample. Examples of valid input files for this application are embedded at the ‘Data Input’ sections to facilitate file formatting and preparation.

DEApp can be used for the analysis of single-factor and multi-factor experiments, even though by default DEApp is used for DE analysis of RNA-Seq data, DEApp can also be used for the identification of differential binding analysis using ChIP-Seq data, and differentially expressed micro RNA analysis using miRNA-Seq data.

After the data is uploaded on the ‘Data Input’ section, the ‘Data Summarization’ panel allows users to set up the cutoff values to filter out genetic features with very low count, as genetic features must present at certain minimal level to provide enough statistical significance for the DE multiple comparison tests. Usually it is recommended to keep genetic features which are expressed in at least one sample out of each factorial group level [11] with a defined number of reads represented by counts per million (CPM) value. By default, the application removes low expression genetic features after alignment with CPM value ≤1 in less than 2 samples. A detailed explanation on how to choose the optimal cutoff values for this step is available in the ‘introduction’ page of the system. Based on the provided cutoff values, a summary of library sizes and normalization factors for each experimental sample, before and after removal of low expression genomic features is displayed on the web interface. The sample’s normalization and multidimensional scaling (MDS) plot are also presented on the web interface to illustrate samples distribution and relationship after filtering out the low expression genomic features. Once this step is completed, the user will be presented with three commonly used methods to perform DE identification.

For a single-factor experiment, the DE analysis can be conducted between any 2 factorial groups of that single-factor; for a multi-factor experiment, the DE analysis can be conducted between any 2 selected groups out of a combination of all group levels. After specifying the group levels, the user will then need to select the parameter cutoffs to determine statistical significance. This includes nominal *p*-value, false discovery rate (FDR) adjusted *p*-value, and fold change (FC). The cutoffs for these parameters can be modified interactively on the web interface for each DE analysis section. The system then will display the dispersion plot, overall DE analysis results, and statistically significant DE results together with a volcano plot interactively corresponding to the specified parameters and cutoff values. Additionally, DEApp also provides a ‘Methods Comparison’ section that enables the comparison and cross-validation of DE analysis results with the implemented analysis methods. A summarized Venn diagram and a table will be presented on the user interface to illustrate the overlapped DE genomic features out of any 2 or all 3 selected analysis methods.

DEApp represents an intuitive alternative to the use of command line commands and scripts, or a basic functionality open source alternative to commercial packages like Partek [12] and CLC Genomics workbench (CLC bio, Aaarhus, Denmark), that are able to offer extensive analytics and sophisticated visualizations for a premium.

The functionality of DEApp can be further expanded to cover complex experiment designs with nested interactions, additive blocking, etc. It will also be possible to expand the automation of further downstream analysis to cover functional annotation and enrichment analysis.

## Conclusion

DEApp enables researchers without sufficient programming experience to perform, evaluate, cross validate, and interactively visualize DE analysis of count-based NGS data easily. This application could potentially expedite the adoption of NGS application in the biomedical research labs.

## Availability and requirements

**Project name**: DEApp**Project home page**: https://yanli.shinyapps.io/DEApp and https://gallery.shinyapps.io/DEApp **Project source code**: https://github.com/yan-cri/DEApp**Operating system**: Platform independent**Programming language**: R (>=3.2) shiny**Other requirement**: Requested R packages including shiny, edgeR, limma, DESeq2 etc.**License**: GPLv2**Any restrictions to use by non-academics**: None

## Abbreviations

DEApp: Differential expression, Analysis Application
NGS: Next generation sequencing
DE: Differential expression
CPM: Counts per million
FDR: False discovery rate
FC: Fold change
